# Cancer cell differentiation heterogeneity and aggressive behavior in solid tumors

**DOI:** 10.3109/03009734.2012.659294

**Published:** 2012-04-19

**Authors:** Annika Jögi, Marica Vaapil, Martin Johansson, Sven Påhlman

**Affiliations:** ^1^Department of Laboratory Medicine, Center for Molecular Pathology, Skåne University Hospital Malmö, Malmö, Sweden; ^2^CREATE Health, Lund University, Malmö, Sweden

**Keywords:** Breast cancer, dedifferentiation, differentiation, EMT, hypoxia, neuroblastoma, prostate cancer, skin cancer, tumor-initiating cells, tumor stem cells

## Abstract

The differentiation stage of tumors is a central aspect in the histopathological classification of solid malignancies. The differentiation stage is strongly associated with tumor behavior, and generally an immature tumor is more aggressive than the more differentiated counterpart. While this is common knowledge in surgical pathology, the contribution of differentiation-related gene expression and functions to tumor behavior is often overlooked in the experimental, tumor biological setting. The mechanisms by which tumor cell differentiation stages are perturbed or affected are poorly explored but have recently come into focus with the introduction.of the tumor stem cell concept. While developmental biologists view the differentiation as a unidirectional event, pathologists and tumor biologists have introduced the concept of dedifferentiation to explain phenotypic changes occurring in solid tumors. In this review we discuss the impact of the tumor cell differentiation stage as used in surgical pathology. We further discuss knowledge gained from exploring the molecular basis of the differentiation and dedifferentiation processes in neuroblastoma and breast cancer, two tumor forms where the tumor cell differentiation concept is used in the clinical diagnostic work and where the tumor stem cell theory has been applied.

## Introduction

The biological meaning of the term differentiation denotes the developmental process whereby cells gradually acquire the capacity for a more specialized function by change of phenotype. While developmental biologists generally view this process as unidirectional, observations in the context of cancer show that the differentiation process can reverse and that cells can dedifferentiate. Despite the fact that classical tumor diagnostics based on surgical pathology and histology for decades have used tumor cell differentiation status as one important aspect to score, evaluate, and communicate tumor aggressiveness, overall experimental tumor biology has over the years not focused on the differentiation processes, but rather studied molecular pathways leading to growth, migration, and cell death. However, the rapid and recent development of stem cell as well as tumor stem cell research have galvanized the study of the differentiation processes in cancer, which has provided insights into the cellular and molecular biological underpinnings of cancer-driven mechanisms leading to changes in the degree of cellular differentiation or dedifferentiation. In this contribution we discuss the impact of the tumor cell differentiation stage on tumor behavior and the use of this important concept in surgical pathology. We further summarize knowledge gained from exploring the differentiation and dedifferentiation processes in two tumor forms, neuroblastoma and breast cancer.

## Differentiation as a prognostic parameter in surgical pathology

In the realm of surgical pathology, the concept of cellular differentiation is made very concrete although used from a different angle and with a somewhat different content than its usage in developmental biology. Apart from performing the obvious task of ruling out/determining whether a tumor is malignant or benign, the pathologist also most often evaluates malignancies aiming to assess their degree of differentiation, implying that neoplastic cells have the potential to slide back along the line of differentiation. The result has prognostic implications where, as a rule, a high degree of differentiation purports a better prognosis than a low degree. Using morphological criteria, the degree of resemblance between the neoplasia and its tissue of origin is gauged. A high degree of differentiation means that the neoplasia is morphologically similar to the native organ, forming neoplastic organoid structures, whereas the opposite is true for a low stage of differentiation, where cells gradually lose the capacity for structural organization and start to display reduced cohesiveness and where the term anaplasia denotes tumor morphology where all similarity with the origin has been lost.

The results from evaluating the degree of differentiation are often presented using a two- or three-tiered scale as exemplified by the common skin malignancy, squamous cell carcinoma (SCC). If a case of SCC grows in an outward (verrucous) fashion, still maintains keratin-forming capacity, has cells with ample cytoplasm, only slight nuclear atypia, and only few mitotic figures, it is regarded as highly differentiated. This means that no signs of invasion below the basal membrane are seen and metastatic disease is rare. On the other side of the scale is the SCC of low differentiation, where cells grow in sheets and with no signs of keratin formation. In these cases the mitotic index is often high, and occasionally immunohistochemistry has to be used to conclude that this cancer indeed is derived from the squamous epithelium. Biologically, these poorly differentiated cancers often invade deeply through the dermis and have a bad prognosis with a propensity for lymph node metastasis. In between these poles are the intermediately differentiated cases of SCC.

Another pertinent example of how differentiation staging is used in modern surgical pathology is the Gleason scoring system developed for prostate cancer. Neoplastic prostate glands are here judged for their capacity to form glandular structures. Grades 1 and 2 are similar to native glands, whereas grade 3 demonstrates glands of reduced diameter growing as separate units in the prostatic tissue, still respecting other glands, malignant as well as benign. Grade 4 heralds that the malignant glands start to coalesce and fuse, whereas grade 5 show malignant cells growing either in sheets or as single cell units, totally devoid of gland-forming capacity. To arrive at the Gleason score, the grade of the most commonly seen cancer (e.g. 3) is added to that of the minority pattern (e.g. 4), which gives a Gleason score of 7. In this case a composite score based on the degree of dedifferentiation is used, which has contributed significantly to the prognostic power of the Gleason grading system.

The morphological correlates of dedifferentiation have been known to pathologists for over a century, but an explanation of the mechanistic factors behind this process has been lacking and unexplored until recent times when the armamentarium of cellular and molecular tumor biology has been deployed to study this phenomenon. A process of interest in this context is the epithelial to mesenchymal transition (EMT). This term describes how epithelial cells phenotypically transdifferentiate towards a more mesenchymal/fibroblastoid/spindle-shaped cell, simultaneously gaining increased capacity for invasiveness and motility. An important question in this context is to what degree EMT equates to dedifferentiation. Addressing this issue, it is important to remember that, by definition, carcinomas develop from epithelial cells. Epithelial cells, however, are not defined at a cellular but at an architectural level, where epithelial cells form multidimensional cohesive cellular sheets of varying thickness resting upon the basal lamina, resulting functionally in immobile cells. The mesenchymal cell, on the other hand, is defined at a cellular level, being spindle-shaped, bipolar, and motile. These cellular categories are the two prototypic cells of chordates (1) from embryogenesis and onwards. Actually, the co-ordinated actions of these dual cell types form the very basis for development of higher life forms, above the level of amphioxi, underscoring their fundamental developmental importance. With this definition in mind the terms dedifferentiation and EMT show a considerable degree of conceptual overlap. Although implicated during invasion, intravasation, and metastasis, clear-cut examples of clinically relevant EMT in cancers have been hard to demonstrate, even if attempts have been made (2). However, as pointed out, dedifferentiated tumors indeed have a considerably worse prognosis, and this category is more often found to invade vascular and neural structures and transgress histological boundaries such as organ capsules, which microscopically recapitulates the *in vitro* data on cells performing EMT.

## Neuroblastoma and tumor cell differentiation

There are few tumor forms that present such a tight link between clinical behavior and tumor cell differentiation stage as the childhood cancer neuroblastoma. Being derived from sympathetic nervous system precursor cells or immature neuroblasts, neuroblastoma cells are arrested at varying stages of differentiation, and, based on histopathology and degree of morphological differentiation, three tumor variants have been defined: 1) the benign ganglioneuromas exclusively containing ganglion-like cells and stroma, 2) ganglioneuroblastomas containing neuroblast-like immature cells with small nuclei and scant cytoplasms intermixed with a smaller or larger proportion of more differentiated tumor cells with larger nucleus and cytoplasm, and 3) neuroblastoma proper with neuroblast-like cells and lacking tumor cells that show apparent signs of morphological ganglionic differentiation. More intriguing, neuroblastomas show one of the highest rates of spontaneous differentiation, a phenomenon first reported in 1927 by Cushing and Wolbach who described a child with a sympaticoblastoma (later termed neuroblastoma) that spontaneously developed into a more differentiated, non-aggressive tumor with a differentiated sympathetic phenotype (3). At the molecular level, the association between high expression of sympathetic ganglionic marker genes and favorable disease was established 80 years later based on global gene expression analyses (4). Through the pioneering work by June Biedler, Robert Seeger, and others, neuroblastoma was one of the first human tumors to be established in culture, and some of these cell lines were shown to have retained the capacity to differentiate along a ganglionic lineage *in vitro* in response to external stimuli such as phorbol esters and retinoic acid (5,6). These findings suggested that aggressive neuroblastomas might become treatable by inducing a differentiation response as part of the treatment protocol. Today 13-cis-retinoic acid at pharmacological levels is used as adjuvant treatment following myeloablative therapy; whether the observed clinical effects are related to retinoic acid-induced changes in the stage of tumor cell differentiation has not been established.

## Neuroblastoma and phenotypic heterogeneity

In a subset of neuroblastomas, tumor cells are organized in lobular structures with zones of necrotic cells in the lobule center (7). In these lobules a neuronal-to-neuroendocrine lineage conversion occurs with the neuroendocrine cells located adjacent to the necrosis (8). The sympathetic nervous system lineage markers used in these studies could not distinguish between sympathetic paraganglionic and sympathetic SIF (small intensely fluorescent) phenotypes. Hence, the exact nature of the neuroendocrine cells detected in lobular neuroblastomas has not been established, although the adrenal chromaffin marker gene *PNMT* is not expressed, ruling out a neuroblastoma-to-pheochromocytoma conversion pathway. These old data exemplify the frequently occurring intra-tumoral phenotypic heterogeneity in solid cancers and do suggest that such heterogeneity is not exclusively a result of mosaicism of genetic aberrations. Our data further imply the existence of intra-tumoral mechanisms regulating the differentiation stage of tumor cells.

## Hypoxia promotes an immature, stem cell-like neuroblastoma phenotype

Concurrently with our report that peri-necrotic neuroblastoma cells differ in phenotype compared to cells located closer to the fibrovascular stroma, the correlation between tumor hypoxia and aggressive disease was demonstrated (9), and the molecular basis for cellular adaptation to hypoxia began to be resolved. Semenza and co-workers identified hypoxia inducible factor HIF-1 (10), which together with HIF-2 is the central transcription factor governing cellular adaptation to hypoxia (11). These dimeric transcription factors have a β-subunit (also called ARNT) in common and an oxygen-sensitive α-subunit unique for HIF-1 and HIF-2, respectively. At hypoxia the α-subunits become stabilized, transported into the nucleus to dimerize with ARNT, and by additional mechanisms activated to induce the transcription of a number of hypoxia-driven genes (12). Based on the potential impact by which the HIF transcription factors can affect the tumor cell phenotype, we asked if hypoxic cells of established neuroblastoma cell lines gained a neuroendocrine phenotype similar to that of peri-necrotic, lobular, neuroblastoma cells. As reported, none of the tested cell lines showed a hypoxia-induced neuronal-to-neuroendocrine lineage shift, but instead hypoxia promoted an immature, neural crest-like phenotype (13,14). As classical neuroblastoma cell lines are all derived from high-stage, most often *MYCN*-amplified tumors, and not tumors of the lobular type described above, our current hypothesis is that we did not use the adequate cells for testing *in vitro* the capacity of lobular neuroblastoma cells to convert into a neuroendocrine cell under hypoxic conditions. The important outcome of these experiments was the observation that hypoxia can dedifferentiate tumor cells, a finding now corroborated in many different tumor forms (15), including breast cancer as described below. Our observation provides one mechanism behind phenotypic heterogeneity in solid tumors and, importantly, highlights how hypoxia can contribute to the aggressive behavior of tumors with an overall low grade of oxygenation.

## HIF-2α-defined pseudohypoxic phenotype and tumor aggressiveness in neuroblastoma

According to well-established models, both HIF-1α and HIF-2α subunits become degraded via the proteasomes' conditions of proper oxygenation (16). However, in clinical samples we found that HIF-2α but not HIF-1α protein was highly expressed in a small subset of neuroblastoma cells close to blood vessels (17-19). In cell lines we could establish that HIF-2 was active at a subphysiological level of oxygenation (5% oxygen) and transcribing known hypoxia-driven genes such as *VEGF*. Importantly, presence of tumor cells with strong immunohistochemical staining for HIF-2α correlates to high clinical neuroblastoma stage and unfavorable outcome (17). A closer examination of the HIF-2α-positive, peri-vascular cells revealed that they are immature and have a neural crest-like phenotype (18). They further express VEGF, and we speculate that this subset of tumor cells actively attracts vascular endothelial cells. Glioma tumor stem/tumor-initiating cells as defined functionally are immature, have neural stem cell properties, and express high HIF-2α protein levels (20). Like the strongly HIF-2α and VEGF-positive neuroblastoma cells, glioma stem cells are located in a peri-vascular niche and express VEGF (20). Thus, these two neutrally derived tumors contain subsets of cells that are immature, and their presence is associated with an aggressive, unfavorable disease. HIF-2 appears to be involved in keeping the stemness of both neuroblastoma and glioma cells, and, as discussed below, HIF-2α expression in breast cancer is also associated to unfavorable disease, suggesting HIF-2α as a potential treatment target in these tumor forms. As shown in neuroblastoma, by down-regulating HIF-2α in stem cell-like cells, sympathetic neuronal differentiation can be induced (21), and we speculate that HIF-2 inhibition could be a strategy to push immature stem cell-like cells into a more differentiated, bulk-like cell population that can be treated by established treatment protocols.

## Breast cancer and tumor cell differentiation

Breast cancer is an additional tumor type where impaired development is an important component of the malignant process and where the tumor cell differentiation stage is used in histopathological tumor grading. The Nottingham grading system, widely employed for diagnosis and prognosis of breast tumors, is based on three parameters: extent of tubule formation, mitosis frequency, and nuclear pleomorphism, where at least the first and last elements are related to differentiation stage. Tubule formation requires presence of differentiated polarized epithelial cells and that these cells orientate in relation to adjacent cells. Thus, the capacity to form tubules is indicative of the differentiation stage of a group of breast cancer cells. In breast cancers cells are unable to distinguish between apical and basal surface and organize themselves into tubule-like organoid structures, have a low stage of differentiation, and receive a high score in the Nottingham grading system. Increased nuclear-to-cytoplasmic ratio is also a marker of low differentiation, and when it is associated with variation in nuclear size between cancer cells the tumor gets a high score also in this element of the Nottingham grading system. A high Nottingham score (8–9, or grade III) is associated with poor prognosis and progressing disease. The relation between differentiation stage and prognosis in breast cancer implies that the understanding of processes of cancer initiation and progression should be paralleled by insight into mechanisms of normal breast development and function.

The tissue origin in breast cancer, the mammary gland, is continuously in a developmental phase in fertile women. Puberty and pregnancy are phases of intense epithelial cell growth, migration, and differentiation. The life cycle of the mammary gland in addition holds phases of epithelial regression and tissue remodeling, i.e. the dramatic involution after lactation and tissue regression after menopause. The involution process involves extensive cell death in the epithelial compartment, intense remodeling of the stromal compartment, and infiltration of inflammatory cells. In this apparently chaotic environment the mammary epithelial tissue stem cells must be preserved to ensure rebuilding of the functional gland in future pregnancies. All these processes of the mammary gland functional cycle, proliferation, differentiation, and regression also occur in each monthly estrous cycle but to a lesser extent. There is a strong correlation between the total number of estrous cycles during lifetime (depending on early menarche, late menopause, and number of child-births) and the risk of breast cancer, implying that stem cell activation and differentiation processes are linked to breast tumorigenesis (22).

**Figure 1.**
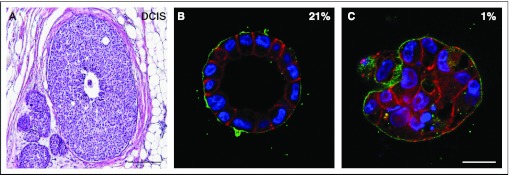
A: Ductal carcinoma *in situ* of the breast, the comedo form, with several cell layers of epithelial cells surrounding a central necrotic area. The inner cell layers, adjacent to the necrosis, show low differentiation with unorganized structures and increased nucleus-to-cytoplasm ratio (scale bar: 500 μm). B: Non-malignant mammary epithelial cells cultured in a three-dimensional differentiation-inducing assay. After 21 days of culture at normoxia (21% O_2_), the mammary epithelial cells (MCF-10A) differentiate into growth-arrested, organized acini structures with polarized cells surrounding a hollow lumen, resembling the *in vivo* mammary gland appearance. The differentiated mammary cells have a polarized expression pattern of proteins, here laminin V (green), and small compact nuclei (blue, actin in red) in a palisade structure. C: At hypoxia (1% O_2_) the MCF-10A mammary epithelial cells fail to arrange into organized structures and appear as cell aggregates without lumen or polarized protein localization. The hypoxic cells have larger nuclei, remain proliferative, and express markers of undifferentiated cell stage—characteristics often seen in breast carcinoma (scale bar: 20 μm).

In breast ductal carcinoma *in situ* (DCIS) with comedo lesions, layers of transformed epithelial cells fill up the ductal space and due to intra-lesional hypoxia a central necrotic core arises (Figure 1A). Cell layers close to the central necrosis are hypoxic and have increasing protein levels of e.g. HIF-1. We have reported that the breast cancer cells close to the necrotic core have a less differentiated phenotype as assessed by histopathological criteria (23). The hypoxic cells have increased nuclear-to-cytoplasmic ratio and decreased expression of markers of differentiation, including estrogen receptor (ER) in ER-positive lesions. Furthermore, small duct-like structures frequently form within DCIS lesions and represent transformed epithelial cells striving to organize themselves into polarized epithelial structures, structures that are rarely encountered in the hypoxic peri-necrotic regions of the DCIS lesions. Thus, intra-lesional hypoxia has direct bearing on two of the elements of the Nottingham grading system, nuclear phenotype and tubule formation. Our hypothesis is that the hypoxic conditions lead to a less differentiated tumor cell with lost ability to form organized and polarized structures. In support of this hypothesis, cells of established human breast cancer cell lines cultured at hypoxic conditions showed down-regulated expression of ER and up-regulation of CK19, indicating that, both *in vitro* and *in vivo*, hypoxic conditions promote a less differentiated breast cancer phenotype (23).

DCIS is an instance of a typical carcinoma *in situ* lesion where the basal membrane has not been breached, meaning that the cancer is non-invasive. Initially regarded as a fairly homogeneous disease divided into non-comedo versus comedo type, DCIS is now seen as a multi-faceted entity where the degree of differentiation within the DCIS lesion has a large impact on the outcome. Cases displaying low differentiation most often progress to invasive carcinoma, whereas cases of high differentiation are less likely to do so. This underscores the importance of assessing differentiation also in pre-invasive cancers. As a whole, DCIS may be seen as an intermediate step in the malignification process of tumors, and the typical hallmark trait of low differentiation in this form of neoplasia is the presence of comedo-type necrosis. This means that the cancer tissue has a necrotic and hypoxic center. This implies that hypoxia might contribute to the conversion of DCIS cells into invading tumor cells. One question we have addressed is whether the observed hypoxic impairment of differentiation in DCIS lesions is restricted to cancer cells. A related question is whether hypoxia can arrest normal breast epithelial cells at an immature differentiation stage and by doing so contribute to onset of tumorigenesis. We have studied the non-malignant immortalized mammary epithelial MCF-10A cells in 3D cultures where these cells form mammary acini with polarized rim cells and evacuated lumen. Hypoxia impaired both polarization and lumen formation as demonstrated in MCF-10A cells (Figure 1B and C) (Vaapil et al., unpublished paper, 25). Furthermore, a fraction of hypoxic MCF-10A cells remained in cell cycle, while the vast majority of the normoxic cells entered a differentiated post-mitotic state. Still the hypoxic structures were smaller, which we could attribute to increased apoptosis. We conclude that hypoxia confers a cancer-like phenotype to the mammary epithelial cells.

It is nowadays well established that intra-tumor hypoxia correlates to a worse prognosis in many tumor types including breast cancer (9). Increased tumor protein levels of HIF-1 and HIF-2, respectively, are also linked to poor patient outcome in breast cancer (reviewed in (26)). In particular, we have shown that HIF-2 protein accumulation correlates to worse breast cancer specific survival and distant metastasis (26). Formation of distant metastases has been attributed to the presence of cancer stem or tumor-initiating cells, and we speculate that HIF-2 is a marker of such cell populations. The nature of the breast cancer stem cell is still far from established; the CD44^+^/CD24^-^ phenotype reported by Al-Hajj et al. (27) does not seem to be the exclusive breast tumor-initiating cell totem. It has not been established that breast tumor-initiating cells have breast stem cell phenotype, and it has lately been implied that stem cell-like cancer cells can arise from more differentiated cells, e.g. through the process of EMT (28). Hypoxia has been shown to induce EMT in tumors (29) and may be one process wherein breast cancer cells with stem cell properties arise. This general differentiation-counteracting effect of hypoxia has direct bearing on tumor aggressiveness, as tumors with immature features are more aggressive than the corresponding differentiated tumors.

## Tumor cell differentiation/dedifferentiation pathways in relation to normal development

**Figure 2.**
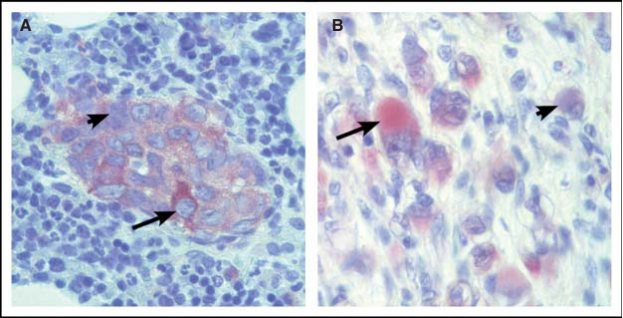
Human neuroblastoma specimens stained for neuron-specific enolase by immunohistochemistry. Sections of a neuroblastoma bone-marrow metastasis (A) and a ganglioneuroma specimen (B), respectively, stained for neuron-specific enolase (*ENO2*) expression. Note that tumor cells differ considerably in neuron-specific enolase levels, both at a more immature (panel A) and at a differentiated (panel B) stage. Arrows show enolase-positive and arrow-heads show enolase-negative tumor cells.

Tumor cells recapitulate morphology and central gene expression profiles of non-malignant cells, which is the basis for histopathological and immunohistochemical classification of solid tumors. The differentiation traits, apparent or delicate, together with location of the primary tumor, determine the histopathological diagnosis. Both at the morphological and molecular levels, the degree or stage of differentiation of the tumor cells is estimated, and depending on the outcome of these estimations tumors are often viewed as being arrested at early or late stages of differentiation. However, whether a given tumor cell differentiation stage reflects a distinct stage during normal development has to our knowledge not been studied in any detail. Based on our own neuroblastoma data, we would claim that this is not the case. While normally developing human sympathetic neuroblasts appear to express a given set of genes in a co-ordinated and repeated fashion between embryos (30), only a subset of the same set of genes is usually expressed in a given tumor, and this subset can differ from one tumor to another and between tumor cells within a tumor as exemplified by the neuron-specific enolase (*ENO2*) expression (in neuroblastoma and ganglioma, the differentiated form of neuroblastoma) (Figure 2) (31). Thus, we would like to claim that differentiation pathways are disorganized in neuroblastoma and that this aberrant differentiation is a tumor characteristic. We hypothesize that this might also be the case in many other tumor forms.

Similarly, when tumor cells dedifferentiate, do they then play back and recapitulate in reverse the developmental stages that once formed them? And when for instance hypoxic cells develop stem cell-like phenotypes, do they indeed become stem cells? These are central questions as they relate to the concept of tumor stem cells or tumor-initiating cells. Without formal proofs and experimental backup, we assume that the stem cell phenotype of tumor cells only mimics that of the cognate stem cell. However, as demonstrated in glioma, the glioma stem cells have the capacity of neural stem cells to give rise to distinct non-glial differentiation lineages (32,33). Thus, while the transcriptome of tumor stem cells may not fully match that of the cognate stem cells, pluripotent tumor cells with stem cell phenotype and capacity probably contribute significantly to the phenotypic heterogeneity seen in solid cancers where the EMT process perhaps is the most extensively studied.
